# Insights into Microalga and Bacteria Interactions of Selected Phycosphere Biofilms Using Metagenomic, Transcriptomic, and Proteomic Approaches

**DOI:** 10.3389/fmicb.2017.01941

**Published:** 2017-10-10

**Authors:** Ines Krohn-Molt, Malik Alawi, Konrad U. Förstner, Alena Wiegandt, Lia Burkhardt, Daniela Indenbirken, Melanie Thieß, Adam Grundhoff, Julia Kehr, Andreas Tholey, Wolfgang R. Streit

**Affiliations:** ^1^Department of Microbiology and Biotechnology, Biocenter Klein Flottbek, Universität Hamburg, Hamburg, Germany; ^2^Bioinformatics Core, University Medical Center Hamburg-Eppendorf, Hamburg, Germany; ^3^Core Unit Systems Medicine, University of Würzburg, Würzburg, Germany; ^4^Division of Systematic Proteome Research and Bioanalytics, Institute for Experimental Medicine, University of Kiel, Kiel, Germany; ^5^Virus Genomics, Leibniz Institute for Experimental Virology, Heinrich-Pette-Institute, Hamburg, Germany; ^6^Molecular Plant Genetics, Biocenter Klein Flottbek, Universität Hamburg, Hamburg, Germany

**Keywords:** microalga–bacteria interaction, phycosphere biofilm, metagenomics, metatranscriptomics, metaproteomics

## Abstract

Microalga are of high relevance for the global carbon cycling and it is well-known that they are associated with a microbiota. However, it remains unclear, if the associated microbiota, often found in phycosphere biofilms, is specific for the microalga strains and which role individual bacterial taxa play. Here we provide experimental evidence that *Chlorella saccharophila, Scenedesmus quadricauda*, and *Micrasterias crux-melitensis*, maintained in strain collections, are associated with unique and specific microbial populations. Deep metagenome sequencing, binning approaches, secretome analyses in combination with RNA-Seq data implied fundamental differences in the gene expression profiles of the microbiota associated with the different microalga. Our metatranscriptome analyses indicates that the transcriptionally most active bacteria with respect to key genes commonly involved in plant–microbe interactions in the Chlorella (Trebouxiophyceae) and Scenedesmus (Chlorophyceae) strains belong to the phylum of the α-Proteobacteria. In contrast, in the Micrasterias (Zygnematophyceae) phycosphere biofilm bacteria affiliated with the phylum of the Bacteroidetes showed the highest gene expression rates. We furthermore show that effector molecules known from plant–microbe interactions as inducers for the innate immunity are already of relevance at this evolutionary early plant-microbiome level.

## Introduction

It is well-known that higher plants have evolved from a distinct phylogenetic branch of algae that includes chlorophytes, charophytes, prasinophytes, glaucophytes, and red algae. Overall, microalgae comprise a highly heterogeneous group of prokaryotic and eukaryotic microorganisms (Baldauf, 2003; Laurin-Lemay et al., 2012; Leliaert et al., 2012, 2016; Ruhfel et al., 2014). Like plants, microalgae are associated with a microbiota, and it is well established that the bacteria play a pivotal role for nutrient uptake and B-vitamin production (e.g., B12, thiamin, biotin, and riboflavin; Warren et al., 2002; Croft et al., 2005; Helliwell et al., 2011; Giovannoni, 2012; Seymour et al., 2017). Additionally, growth promoting factors, chelators, and phytohormones are produced by associated bacteria and can support algal growth (Stirk et al., 2002; Tang et al., 2010).

In contrast to the relatively high biodiversity observed in native plant microbiomes (i.e., rhizospheres), recent research has provided evidence that microalga are of in general colonized by a less diverse microbial community (i.e., phycospheres). In general, fewer than 30 bacterial isolates on a species level are affiliated with these alga–bacterial phycosphere biofilms. These publications imply that within the microalga microbiomes frequently bacteria affiliated with the phyla of the Bacteroidetes, the α-, β-, 𝜀-, and the γ-Proteobacteria can be found (Jones et al., 2007; Krohn-Molt et al., 2013; Mendes et al., 2013; Knief, 2014; Knack et al., 2015; Ramanan et al., 2016). Many of these studies have used microalga obtained from culture collections in which the alga microbiomes have been cocultivated with the alga often over many decades. Because of the long-term cocultivation it can be assumed that presumably well-adapted microbial communities have been established under these conditions. These communities are most likely less diverse and differ in contrast from those observed in the water zone of marine or aquatic phytoplankton (Buchan et al., 2014; Seymour et al., 2017).

Currently, it is not well known to which extend aquatic microalga–bacteria interactions are specific or which algal factors affect the selection for the bacterial colonizers. Furthermore, it is unknown, whether microalgae have established immune systems similar to higher plants to respond to the bacterial colonization and infection. Within this context it is well known that plants have evolved two distinct immune systems. The evolutionary older system relies on the recognition of common microbe-associated molecular patterns. These are general molecules like flagellins, exoenzymes, cold shock proteins, elongation factor Tu (EF-Tu), modified carbohydrates and lipids among others. The second, evolutionary younger system is designated as **e**ffector-**t**riggered **i**mmunity (ETI) with the signals being perceived by variable cognate plant immune responsive receptor proteins (He et al., 2006; Jones and Dangl, 2006; Boller and He, 2009).

Within this framework, we addressed the following questions in the current study: Can we identify specific bacterial communities associated with microalga affiliated with the Trebouxiophyceae, Chlorophyceae, and Zygnematophyceae using material from strain collections that has been cultivated over long-time periods? Who are the transcriptionally most active bacteria in these communities on a genome, transcriptome, and proteome level; and are the main bacterial transcripts and secretome-proteins the same as those known as elicitors to plant innate immune system? To answer these questions we have systematically characterized the microbiomes of selected Trebouxiophyceae, Chlorophyceae, and Zygnematophyceae obtained from the “Microalgae and Zygnematophyceae Collection Hamburg” (MZCH).

Further, detailed and deep metagenome and metatrans criptome analyses identified shared and host-specific transcripts in microalgal microbiomes. This way, we have produced a detailed microbiome dataset for *Chlorella saccharophila, Scenedesmus quadricauda*, and *Micrasterias crux-melitensis*. These analyses will help to improve our understanding of possible plant–microbe interactions on an early evolutionary level.

## Materials and Methods

### Isolation of Microalgae Strains

For the isolation of novel unicellular alga, we used a low energy laser trap microscope (PALM MicroTweezers, Zeiss, Germany). This microscope is equipped with optical tweezers and a vacuum suction device. We have been using this to isolated individual microalga from environmental freshwater samples of a pond of the “Botanical Garden” (Hamburg, Germany, latitude: 53.5667, longitude: 9.9833).

### Microorganisms Used in This Study and Cultivation Media

Microalga isolates were either obtained from the MZCH^1^ and newly isolated. The following isolates were cocultured for a minimum of 10 years in the strain collection: *C. saccharophila* (MZCH 10155), *Chlorella vulgaris* pv minima HH (MZCH 10162), *Chlorella zofingiensis* (MZCH 10156), *Scenedesmus acuminatus* (MZCH 10102), *Scenedesmus carinatus* (MZCH 10103), *S. quadricauda* (MZCH 10104), *Micrasterias furcata* (MZCH 75), *M. crux-melitensis* (MZCH 98), and *Micrasterias muricata* (MZCH 125). Recently newly isolated strains are: *Chlamydomonas* sp. HH 10201, *Chlamydomonas* sp. HH 10204, *Acutodesmus* sp. HH 10202, *Desmodesmus* sp. HH 10203, *Scenedesmus* sp. HH 10205, and *Scenedesmus* sp. HH 10206.

Cultivation of the individual alga species was done as follows: for the cultivation of *C. saccharophila* (MZCH 10155), we used an Ld+OHC-media with minor modifications (Aaronson and Baker, 1959). The medium was composed of 0.05 g/l Ca(NO_3_)_2_ × 4H_2_O, 0.05 g/l MgSO_4_ × 7H_2_O, 0.003 g/l K_2_HPO_4_, 0.25 ml micronutrient solution [stock solution: 0.5 g/l FeSO_4_ × 7H_2_O, 1.0 g/l H_3_BO_3_, 0.5 g/l MnCl_2_ × 4H_2_O, and 5.0 g/l titriplex III (EDTA)], 1 ml/l vitamin solution (stock solution: 0.1 g/l thiamin × HCl, 0.01 g/l biotin, 0.002 g/l cyanocobalamin, and 0.001 g/l niacin), and 10 ml/l OHC solution (stock solution: 100 g/l D-glucose, 100 g/l bacto-tryptone, 100g/l liver extract, and 100g/l yeast extract). The cultivation of *C. vulgaris* pv minima HH (MZCH 10162), *C. zofingiensis* (MZCH 10156), *Scenedesmus* sp. HH 10205, and *Scenedesmus* sp. HH 10206, was carried out in Kessler and Czygan-media with minor modifications (Kessler and Czygan, 1970). The media contained 0.81 g/l KNO_3_, 0.0015 g/l CaCl_2_ × 2H_2_O, 0.25 g/l MgSO_4_ × 7H_2_O, 0.47 g/l NaCl, 0.47 g/l NaH_2_PO_4_, 0.36 g/l Na_2_HPO_4_ × 2H_2_O, and 1.0 ml micronutrient solution [stock solution: 0.6 g/l FeSO_4_ × 7H_2_O, 0.05 g/l H_3_BO_3_, 0.05 g/l MnCl_2_ × 4H_2_O, 0.8 g/l titriplex III (EDTA), 0.02 g/l ZnSO_4_ × 7H_2_O, and 0.02 g/l (NH_4_)_6_Mo_7_O_24_ × 4H_2_O]. *S. quadricauda* (MZCH 10104), *Chlamydomonas* sp. HH 10201, and *Acutodesmus* sp. HH 10202 were cultivated in BG11 (Stanier et al., 1971; Rippka et al., 1979). This media consists of 0.038 g/l CaCl_2_ × 2H_2_O, 0.075 g/l MgSO_4_ × 7H_2_O, 0.041 g/l K_2_HPO_4_ × 3H_2_O, 1.50 g/l NaNO_3_, 0.04 g/l Na_2_CO_3_, 0.006 g/l citric acid, 0.006 g/l ferric ammonium citrate, 0.001 g/l triplex III (EDTA), 1.0 ml micronutrient solution [stock solution: 0.079 g/l CuSO_4_ × 5H_2_O, 0.049 g/l CO(NO_3_)_2_ × 6H_2_O, 2.86 g/l H_3_BO_3_, 1.81 g/l MnCl_2_ × 4H_2_O, 0.39 g/l Na_2_MoO_4_ × 2H_2_O, and 0.222 g/l ZnSO_4_ × 7H_2_O], and 4.78 g/l HEPES buffer. For thei cultivation of *S. acuminatus* (MZCH 10102), *S. carinatus* (MZCH 10103), *M. crux-melitensis* (MZCH 98), *M. furcata* (MZCH 75), *M. muricata* (MZCH 125), *Chlamydomonas* sp. HH 10204, and *Desmodesmus* sp. HH 10203 we used a Woods Hole MBL-media (Stein, 1979). This media contains 0.037 g/l CaCl_2_ × 2H_2_O, 0.037 g/l MgSO_4_ × 7H_2_O, 0.009 g/l K_2_HPO_4_, 0.013 g/l NaHCO_3_, 0.085 g/l NaNO_3_, 0.028 g/l Na_2_SIO_3_ × 9H_2_O, 1.0 ml micronutrient solution (stock solution: 0.01 g/l COCl_2_ × 6H_2_O, 0.01 g/l CuSO_4_ × 5H_2_O, 3.15 g/l FeCl_3_ × 6H_2_O, 0.18 g/l MnCl_2_ × 4H_2_O, 4.36 g/l Na_2_EDTA, 0.006 g/l Na_2_MoO_4_ × 2H_2_O, 0.02 g/l ZnSO_4_ × 7H_2_O), 1 ml/l vitamin solution (stock solution: 0.0001 g/l thiamin × HCl, 0.0005 g/l biotin, 0.0005 g/l cyanocobalamin, and 0.001 g/l niacin), and 0.5 g/l HEPES buffer. The pH value was adjust to 7.0–7.2.

The microalgae were cultivated at 22°C in liquid medium with a final concentration of 4% CO_2_ at a natural light intensity for 14 h per day and a dark period of 10 h per day.

### Scanning Electron Microscopy

Scanning electron microscopy (SEM) was performed as previously published (Krohn-Molt et al., 2013). Therefore, samples were fixed in paraformaldehyde (1%) and glutaraldehyde (0.25%), dehydrated by ascending alcohol series and dried at the critical point with Balzers CPD 030 Critical Point Dryer (BAL-TEC, Schalksmühle, Germany). After coating samples with gold using a sputter coater SCD 050 (BAL-TEC), scanning electron micrographs were taken with a LEO 1525 (Zeiss, Germany).

### Total Bacterial DNA Extraction from Microalgae Samples

Samples were taken from three different microalga: *C. saccharophila* (MZCH 10155), *S. quadricauda* (MZCH 10104) and *M. crux-melitensis* (MZCH 98). At the time of sampling, the microalgae were under steady growth conditions. The used samples were collected at the same photoperiod of the hosts, after 7 h of a daylight period under stable growth condition.

For metagenome sequencing and PCR amplification, total nucleic acids were extracted from the samples using a previously published enzymatic cell lysis protocol (Krohn-Molt et al., 2013), including a sonification (30 s, amplitude 60%, cycle 0.5) and centrifugation step (5 min, 700 × *g*) at the beginning, to separate the bacteria from the microalga. Concentration and purity of DNA was analyzed using a Nanodrop ND-2000 instrument (PEQLAB Biotechnologie GmbH, Erlangen, Germany).

### Analysis of 16S rRNA Genes of Alga-Associated Bacteria and Phylogenetic Analysis of the Alga

For the phylogenetic characterization of the alga-associated microbial community, genes were amplified using an amplicon barcoded sequencing protocol for MiSeq platforms. Variable regions V3 and V4 of bacteria/archaeal genes were amplified using the primer set 515F/806R (515F: 5′-AAT GAT ACG GCG ACC ACC GAG ATC TAC ACT ATG GTA ATT GTG TGC CAG CMG CCG CGG TAA-3′ and 806R: 5′-CAA GCA GAA GAC GGC ATA CGA GAT (barcode) AGT CAG TCA GCC GGA CTA CHV GGG TWT CTA AT-3′ each reverse primer contains different barcode sequences) as previously published (Caporaso et al., 2012; Klindworth et al., 2012).

The PCR contained 100 ng of template DNA/μl, 0.2 mM of each of the four deoxynucleoside triphosphates, 1.5 mM MgCl_2_, 1 μM (each) primer, and 2.5 U of Taq DNA polymerase. PCR cycling conditions were as follows: initial denaturation at 94°C for 3 min, followed by 34 cycles of denaturation at 94°C for 45 s, annealing at 50°C for 60 s, and extension at 72°C for 90 s. The final extension was conducted at 72°C for 5 min. Negative controls were performed with H_2_O instead of template DNA. The obtained PCR products were purified via Gel/PCR DNA Fragments Extraction Kit (Geneaid Biotech, Taiwan) as recommended by the manufacturer. Three separate PCRs were conducted for each sample. The amplified genes were sequenced and analyzed on an Illumina MiSeq following the manufacturer’s instructions. Sequencing was performed in 2 × 151 bp paired-end mode using the MiSeq Reagent Kit v3. For the analysis, Illumina’s “16S Metagenomics” workflow (Analysis software version 2.6.2.3) was executed on-instrument using default settings. The workflow’s classification step is based on Illumina’s proprietary algorithm “ClassifyReads” and a modified version of the Greengenes Database (v.13_5)^2^.

For phylogenetic characterization of the strain collection species: *C. saccharophila* (MZCH 10155), *C. vulgaris* pv minima HH (MZCH 10162), *C. zofingiensis* (MZCH 10156), *S. acuminatus* (MZCH 10102), *S. carinatus* (MZCH 10103), *S. quadricauda* (MZCH 10104), *M. furcata* (MZCH 75), *M. crux-melitensis* (MZCH 98), *M. muricata* (MZCH 125), and the new isolates: *Chlamydomonas* sp. HH 10201, *Chlamydomonas* sp. HH 10204, *Acutodesmus* sp. HH 10202, *Desmodesmus* sp. HH 10203, *Scenedesmus* sp. HH 10205, and *Scenedesmus* sp. HH 10206, the 18S/ITS/26S regions were amplified using oligonucleotide primers with an additional sequencing adapter binding site (M13for-18S/AU500af: 5′-TTG TAA AAC GAC GGC CAG TGG CGC TAC ACT GAT GTA TTC AA-3′ and M13rev-28B: 5′-GGA AAC AGC TAT GAC CAT GAG GTC CGT GTT TCA AGA CGG G-3′ (Friedl, 1996; Kovarik et al., 2005; Lindstrom and Hanic, 2005; Helms et al., 2007). PCR mixtures contained 100 ng of template DNA/μl, 0.2 mM of each of the four deoxynucleoside triphosphates, 1.5 mM MgCl_2_, 1 μM (each) primer, and 2.5 U of Taq DNA polymerase. Thermocycling conditions included 45 s of denaturation at 94°C, 45 s of primer annealing at 58°C, and 1 min 30 s of primer extension at 72°C. This cycle was repeated 34 times. The fragment was sequenced with automated ABI377 technology following the manufacturer’s instructions.

### Bacterial Metagenome Sequencing, *De Novo* Assembly and Binning

Sequencing of metagenomic DNA was performed on the HiSeq 2500 platform using Rapid SBS chemistry (Illumina, San Diego). Libraries were constructed applying the NEBNext^®^ Ultra^TM^ DNA Library Prep Kit for Illumina (New England Biolabs) according to the manufacturer’s protocol. Each sample was sequenced on a single lane of a HiSeq 2500 rapid paired-end run (2 × 250 cycles). Sequencing adapters from the 3′-end of sequencing reads and low quality sequences (Phred quality score below 54) were removed using Trimmomatic (Bolger et al., 2014). Trimmed reads with a length of at least 35 bp were assembled using IDBA-UD (Peng et al., 2012). The resulting contigs were binned using MaxBin (Wu et al., 2014).

Taxonomic profiling of metagenome contigs and bins was performed using AMPHORA2 using a universal marker set of 31 protein encoding phylogenetic marker genes (*dnaG, frr, infC, nusA, pgk, pyrG, rplA, rplB, rplC, rplD, rplE, rplF, rplK, rplL, rplM, rplN, rplP, rplS, rplT, rpmA, rpoB, rpsB, rpsC, rpsE, rpsI, rpsJ, rpsK, rpsM, rpsS, smpB*, and *tsf*; Wu and Scott, 2012). NCBI taxonomy IDs were mapped to phylogenetic lineages given by AMPHORA2.

For the sequences’ functional characterization, we used the IMG/MER pipeline and tools. To further analyze the biological processes linked to the individual genes and open reading frames the KEGG (Nakaya et al., 2013), the COG (Tatusov et al., 2001), and the Pfam (Finn et al., 2008) databases were employed.

### Bacterial RNA Extraction and Sequencing

Triplicates of the stationary growth phase, and after 7 h of a daylight period, of the microalgae–bacteria cultures were sonicated (30 s, amplitude 60%, cycle 0.5) and centrifuged at 700 × *g* for 3 min, to separate the bacteria from the microalga. In a second step, the supernatant (contains the bacteria) were again centrifuged at 11,000 × *g* for 5 min. To extract the total RNA, we used a hot phenol method with minor modifications (Aiba et al., 1981). Therefore, the bacterial culture was mixed with 25 ml ice-cold killing buffer (20 mM Tris–HCl, 5 mM MgCl_2_, pH 7.5), centrifuged at 4,000 × *g* for 10 min at 4°C and shock frozen in liquid nitrogen. Cells were resuspended in 125 μl ice-cold 300 mM sucrose/10 mM sodium acetate solution (pH 5.2) and mixed with 125 μl of 2% (w/v) SDS/10 mM sodium acetate (pH 5.2). The cells were incubated for 90 s at 65°C. The next step, the supplementation of 400 μl hot phenol was followed by an incubation time of 3 min at 65°C with a brief mixture of the suspension every minute. The suspension was shock frozen in liquid nitrogen and spun down at 11,000 × *g* for 10 min at RT, the supernatant was mixed again with 400 μl hot phenol and the steps were repeated. Afterward, 400 μl of phenol/chloroform/isoamyl alcohol (25:24:1 v/v) was added to the supernatant and spun down. This step was repeated, followed by the addition of 400 μl chloroform/isoamyl alcohol (24:1 v/v). After a further centrifugation step at 11,000 × *g* for 2 min, 40 μl 3 M sodium acetate (pH 5.2) and 1 ml 100% (v/v) EtOH were added to the supernatant. The RNA precipitated overnight at -20°C. Afterward, the RNA was spun down for 20 min at 4°C, washed twice with 70% (v/v) EtOH, dried and dissolved in 200 μl DEPC-treated H_2_O. The removal of simultaneously extracted DNA was achieved by using the RTS DNase^TM^ Kit (MO BIO Laboratories Inc., Carlsbad, United States). The RNA was cleaned and concentrated via the RNA Clean & Concentrator^TM^-5 Kit (Zymo Research, Irvine, United States). Concentration and quality of the total RNA was measured by a Nanodrop ND-2000 instrument (PEQLAB Biotechnologie GmbH, Erlangen, Germany) and verified on a 1.2% formaldehyde-agarose gel. The remaining transcripts were used for cDNA library construction by Vertis Biotechnologie AG, Germany^3^. Libraries for Illumina sequencing of cDNA were constructed by Vertis Biotechnology AG, Germany (see text footnote 3), as described previously for eukaryotic microRNAs but omitting the RNA size-fractionation step prior to cDNA synthesis (Berezikov et al., 2006). Equal amounts of RNA samples were poly(A)-tailed using poly(A) polymerase. Then, the 5′-triphosphates were removed by applying tobacco acid pyrophosphatase resulting in 5′-monophosphate. Afterward, a RNA adapter was ligated to the 5′-phosphate of the RNA. First-strand cDNA was synthesized by an oligo(dT)-adapter primer and the M-MLV reverse transcriptase. In a PCR-based amplification step using a high fidelity DNA polymerase, the cDNA concentration was increased to 20–30 ng/μl. A library-specific barcode for multiplex sequencing was part of a 3′-sequencing adapter. The following adapter sequences flank the cDNA inserts: TrueSeq Sense primer 5′-AAT GAT ACG GCG ACC ACC GAG ATC TAC ACT CTT TCC CTA CAC GAC GCT CTT CCG ATC T-3′ and TrueSeq Antisense NNNNNN primer (NNNNNN = 6n barcode for multiplexing) 5′-CAA GCA GAA GAC GGC ATA CGA GAT-NNNNNN-GTG ACT GGA GTT CAG ACG TGT GCT CTT CCG ATC (dT25)-3′. The libraries were sequenced on an Illumina HiSeq 2500 in single-end mode with 100 cycles.

### Processing and Analysis of RNA-Seq Reads

Reads in FASTQ format were trimmed with a cut-off Phred score of 20 by the program fastq_quality_trimmer from FASTX toolkit^4^. The following steps were performed with the tool READemption (Förstner et al., 2014). The poly(A)-tail sequences (introduced in the library preparation) were removed and a size filtering step was performed that eliminated reads shorter than 12 nt. The remaining reads were mapped to the reference metagenome sequences, i.e., the assemblies of the bacterial communities using segemehl (Hoffmann et al., 2009).

The quantification of read per annotated feature as well as the calculation of RPKM (Reads Per Kilobase per Million) value was conducted with READemption’s subcommand “gene_quanti.”

The relative occurrence frequency of each COG in expressed genes in comparison to all genes found in the metagenomes was evaluated by performing a Fisher’s exact test. COGs with a *p*-value (adjusted by the Benjamini–Hochberg procedure) less than 0.05 were considered as enriched. The source code of the scripts for the computational analysis of the RNA-Seq data and the COG enrichment analysis are deposited at Zenodo^5^.

### Secretome Preparation and LC-MS/MS Analysis

The samples of the *S. quadricauda* (MZCH 10104) culture were collected in the stationary growth phase of cultivation, after 7 h of a daylight period. Triplicates of the supernatant were concentrated (1:10) via a Vacuum Concentrator (Eppendorf, Hamburg, Germany). For purification, we used the Thermo Scientific Pierce C18 Spin Columns following the manufacturer’s instructions (Thermo Fisher Scientific, Waltham, United States). The exopolymeric substances secreted by the microorganisms were analyzed concerning their content of expressed proteins. The lyophilized samples were sent to the “Systematic Proteomics and Bioanalytics” center in Kiel, Germany^6^. Each sample was re-dissolved in 20 μl 50 mM ammonium bicarbonate buffer, pH 7.4 (ABC). 15 μl of each sample were mixed with 15 μl 4 M guanidine hydrochloride in ABC. Disulfides were reduced in 10 mM 1,4-D/L-dithiothreitol (60 min at 56°C). Alkylation was performed by addition of iodoacetamide to a final concentration of 55 mM (incubation in the dark at room temperature for 60 min). For enzymatic digestion, the solutions were incubated overnight with trypsin, purified by SPE (C_18_ ZipTips), dried by applying vacuum in a SpeedVac (Eppendorf, Concentrator plus) and resuspended in 20 μl 3% acetonitrile/0.05% formic acid for triplicate LC-MS/MS analysis. Peptide separation was performed on a Dionex Ultimate 3000 UHPLC system equipped with an Acclaim^®^ PepMap 100 analytical column (2 μm, 100 Å, 75 μm × 500 mm) coupled online to a Q Exactive Plus. Chromatographic separation was performed with 0.05% formic acid as buffer A and 80% ACN/0.04% FA as buffer B with following gradient: 0 min, 5% B; 5 min, 5% B; 95 min, 70% B; 100 min, 95% B; 110 min, 95% B; 110.1 min, 5% B; 120 min, 5% B. The flow-rate was set to 300 nl/min and the column oven temperature to 45°C. Mass Spectrometry acquisition was performed in positive mode on a Thermo Q Exactive Plus Orbitrap. For MS/MS, the 10 most intensive ions were fragmented by HCD (normalized collision energy: 25, full scan MS: 300–2,000 *m/z*, resolution 70,000). Data analysis, using the Sequest search algorithm, was performed on Proteome Discoverer against a FASTA databases containing the metagenome data of *S. quadricauda* (MZCH 10104) and common impurities.

### Sequences Obtained and GenBank Submissions

During this study, generated raw sequence data have been deposited to the European Nucleotide Archive under the BioProject number PRJEB15376^7^.

Assemblies of the three microalgae metagenomes are available via IMG/MER^8^ using the IMG ID 3300008885 for the metagenome analysis of bacterial community of *C. saccharophila* (MZCH 10155), the IMG ID 3300005759 for the analysis of the bacterial community of *S. quadricauda* (MZCH 10104), and the IMG ID 3300008886 for the analysis of the bacterial community of *M. crux-melitensis* (MZCH 98).

The obtained 18S/ITS/26S rRNA gene sequences have been deposited in the GenBank database under accession numbers: KX525234–KX525236, MF326553–MF326558.

## Results

### Population Structure of the Alga and Alga-Associated Bacterial Community

Previous studies have shown that microalga obtained from strain collections are associated with various bacterial communities with a rather low diversity, and that these bacteria can form tight phycosphere biofilms (Krohn-Molt et al., 2013; Mendes et al., 2013; Knief, 2014; Knack et al., 2015).

**FIGURE 1:**
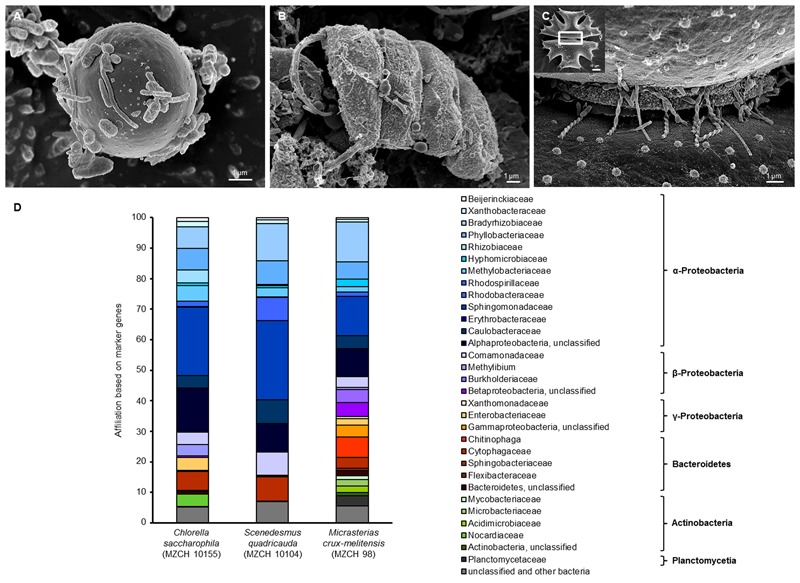
Scanning electron micrograph (SEM) and phylogenetic analysis of **(A)** *Chlorella saccharophila* (MZCH 10155), **(B)** *Scenedesmus quadricauda* (MZCH 10104), and **(C)** *Micrasterias crux-melitensis* (MZCH 98) and the associated microbiota. In panel **(C)**, the white boxed area indicates the area the larger image was taken from; scale bars of 1–2 μm are indicated in the images (REM LEO 1525, 5.00 kV). **(D)** Phylogenetic analysis of bacteria attached to *Chlorella saccharophila* (MZCH 10155), *Scenedesmus quadricauda* (MZCH 10104), and *Micrasterias crux-melitensis* (MZCH 98). The affiliation based on 31 bacterial marker genes by the use of metagenomic datasets analyzed via Amphora2.

In our study, SEM confirmed a tight adherence of relatively few bacteria to the different alga surfaces. In addition, the images also indicated that the cells produced in part net like structures (**Figures 1A–C**). To further analyze these fascinating microbial communities for certain alga species 16S rRNA gene amplicon, and deep metagenome sequencing was initiated.

For the 16S rRNA amplicon sequences analysis, we assigned a minimum of 550,000 reads for each sample. The clustering of bacterial 16S rRNA gene amplicons was done at a 97% similarity level. In general, the analyses resulted in the identification of 7–22 operational taxonomic units (OTUs) per strain and 261 OUTs for the pond (located in the “Botanical Garden” of the University of Hamburg) from which the new strains were isolated. Interestingly, the results of the recently isolated microalga from the pond showed no specificity (Supplementary Figure S1A). In our view, this finding supported the concept that the long-term cultivation has resulted in the selection of distinct bacterial communities. Therefore, we have focused our experiments on the analysis of selected *Chlorella, Scenedesmus*, and *Micrasterias* strains from the “MZCH” (Supplementary Figure S1B). The three analyzed *Micrasterias* species revealed the lowest diversity with only 7–9 OTUs, while the *Chlorella* and *Scenedesmus* microalgae species revealed in general higher numbers of OTUs ranging from 11 to 22. Within this framework, the taxonomic classification of the bacterial 16S rRNA amplicons indicated that the obtained sequences were mainly affiliated with the phyla of the α-, β-Proteobacteria, and the Bacteroidetes. The main bacterial families observed were affiliated with the Comamonadaceae, Caulobacteraceae, Chitinophagaceae, Flexibacteraceae, and Sphingomonadaceae, and included also unclassified microorganisms (Supplementary Figure S1B and **Figure 1D**). While in the *Scenedesmus* phycospheres the Comamonadaceae appeared to be the dominant family present in all samples, no clear pattern was visible for the *Chlorella* phycospheres. In contrast, in the analyzed microbiomes of the *Micrasterias* isolates, the Sphingomonadaceae (α-Proteobacteria), appeared to be the dominant family. No archaeal OTUs were observed in any of the samples.

In general, it is well known that 16S rRNA amplicon sequencing can result in a biased data analysis (Klindworth et al., 2012; Schirmer et al., 2015; Fischer et al., 2016). Thus, to verify the above made findings, we were interested in metagenome-based analyses. Therefore, in addition to amplicon sequencing, a large dataset of metagenomic DNA sequences was produced for the microbiomes of *C. saccharophila, S. quadricauda*, and *M. crux-melitensis*. For each of the three analyzed microbiomes, we generated a minimum of 567 mio reads, which were assembled to 174 Mb of DNA for *C. saccharophila*, 162 Mb of contiguous DNA for *S. quadricauda*, and 268 Mb for *M. crux-melitensis* (**Supplementary Table S1**).

To further analyze the dataset, we employed the AMPHORA2 software, which uses 31 conserved bacterial proteins as phylo genetic markers (Wu and Scott, 2012). A total of 2,800 marker genes were identified and classified in our metagenome datasets. The analysis confirmed largely the 16S rDNA amplicon results with minor differences on the family level. For the *C. saccharophila* metagenome, we identified mainly Sphingomonadaceae, Caulobacteraceae, Comamonadaceae, and unclassified bacteria. The *S. quadricauda* microbiome was also affiliated with members of the Sphingomonadaceae, Bradyrhizobiaceae, Phyllobacteriaceae, Caulobacteraceae, Comamonadaceae, and unclassified bacteria. The main colonizers of the *M. crux-melitensis* microbiome appeared to be Bradyrhizobiaceae, Sphingomonadaceae, Phyllobacteriaceae, Chitinophaga, and bacteria affiliated with the phylum of the Bacteroidetes (**Figure 1D**).

### Metabolic Potential of the Microalga Microbiomes

For the functional analyses, our metagenome data were analyzed using Pfam, COG, and KEGG databases. A total of 236,273 possible proteins were identified for the bacterial community of *C. saccharophila*, 270,387 for the microbial metagenome of *S. quadricauda*, and 349,091 for the microbiome of *M. crux-melitensis*.

#### General Pathway Analyses of the Alga Microbiomes Reveal Great Metabolic Flexibility

The COG and KEGG analyses showed that the metabolic and catabolic potential of the bacterial community living with the used microalgae are highly diverse and flexible (**Table 1**). Bacteria associated with the algae were mainly heterotrophs and metabolizing a wide range of carbon and energy sources. Genes for many of the classical pathways linked to the degradation of biopolymers (e.g., polysaccharides, proteins, cellulose, and lipids) but also for the degradation of aromatic compounds were present. Overall the degradative capabilities were quite similar in the three different analyzed microbiomes.

**Table 1:** Key features observed in the bacterial metagenome of *Chlorella saccharophila* (MZCH 10155), *Scenedesmus quadricauda* (MZCH 10104), and *Micrasterias crux-melitensis* (MZCH 98) using a COG-based analysis.

	*Chlorella*	*Scenedesmus*	*Micrasterias*
	*saccharophila*	*quadricauda*	*crux-melitensis*
	(MZCH 10155)	(MZCH 10104)	(MZCH 98)
Energy production and conversion	6.58	6.05	6.75
General function prediction only	10.56	8.97	10.56
General DNA/RNA metabolism	6.04	6.99	5.86
Cell division and biogenesis	5.89	7.38	6.52
Amino acid metabolism	11.67	8.14	10.53
Carbohydrate metabolism	6.68	5.55	6.49
Lipid metabolism	6.02	4.92	5.87
Metabolism of cofactors, vitamins, and secondary metabolites	9.11	7.95	8.98
Transport mechanisms and secretion system	7.99	7.43	7.98
Cell motility, extracellular structures, and EPS-associated proteins	1.71	2.34	2.16
Signal transduction mechanisms	4.26	5.75	4.97
Defense mechanisms	2.34	2.18	2.66
Transcription	7.02	6.41	6.73
Translation and posttranslational modification	8.74	10.70	8.74
Function unknown	5.38	9.25	5.20

*Data shown in % of all hits.*

The metagenome sequences further suggest that the microbial life style in the three different bacterial metagenomes included most likely aerobic as well as microaerobic growth. Further, genes needed for nitrate respiration and bacterial photosynthesis were observed. Another important trait is the biosynthesis of cofactors and vitamins. B-group vitamins, like thiamin (B1), cobalamin (B12), and biotin are in part essential for microalgae (Warren et al., 2002; Croft et al., 2005; Helliwell et al., 2011; Giovannoni, 2012; Seymour et al., 2017). The metabolic and catabolic analysis of our different data sets suggested that all biosynthesis pathways for vitamin B12, biotin, and thiamin were present in all microbiomes, often in multiple copies.

#### Microbiomes Encode for Many Different Secretion Systems

Plant-associated bacteria interact with their hosts through multiple secretion systems and by releasing specific effector molecules into their host plants (Jones et al., 2007; Knief, 2014). Overall, the highest number of secretion-related genes (per 100 Mb) was observed in the *Micrasterias* (837) microbiome, followed by *Chlorella* (629) and *Scenedesmus* (287). Our analyses revealed that the three microalga microbiomes mainly differed with respect to the frequency and occurrence of the type II, IV, and VI secretion systems (**Table 2**). Interestingly only in the *Micrasterias* microbiome few genes for type III secretion system were identified. These systems are well known to play essential roles in plant–microbe interaction and infection or symbiosis-related processes (Alvarez-Martinez and Christie, 2009; Nivaskumar and Francetic, 2014; Zoued et al., 2014). In addition, main differences existed in the number of genes coding for ABC transporters (**Table 2**).

**Table 2:** Key features of possible inter-kingdom-interaction in the bacterial metagenomes of *Chlorella saccharophila* (MZCH 10155), *Scenedesmus quadricauda* (MZCH 10104), and *Micrasterias crux-melitensis* (MZCH 98), using KEGG- and COG-based analysis.

	*Chlorella*	*Scenedesmus*	*Micrasterias*
	*saccharophila*	*quadricauda*	*crux-melitensis*
	(MZCH 10155)	(MZCH 10104)	(MZCH 98)
**Secretion systems**			
Type I	43	29	61
Type II	72	41	149
Type III	–	0.6	15
Type IV	210	72	263
Type VI	76	–	50
Sec (secretion) system	185	120	249
Sec-independent	43	25	50
**Transporter**			
ABC transporter	3,553	1,501	4,185
**Signal transduction**			
Two-component system	1,845	1,070	2,611
Autoinducer (AI-1 and AI-2)	10	4	14
Nodulation (Nod) factor	16	4	12
Flagellin	42	44	53
**Carbohydrate and lipid modification**			
Glycosyl hydrolases	115	73	95
Sulfotransferase	45	28	48
Methyltransferases	13	–	–
Deacetylase	129	71	155
Esterase/lipase	907	288	1,245

*Data shown in total number of hits per 100 Mb.*

Overall, cell–cell communication is of high importance for plant root colonization and biofilm formation (Rodríguez-Navarro et al., 2007; Krysciak et al., 2014). Therefore, we analyzed the number of genes involved in the biosynthesis of autoinducer molecules (AI-1 and AI-2). Not more than 14 genes coding for AI biosynthesis proteins per 100 Mb were observed (**Table 2**).

Furthermore, Nod factors play a significant role in bacterial plant interaction and usually are of relevance for symbiotic infections (Rodríguez-Navarro et al., 2007). Surprisingly, in each of the systems, we observed a small number (4–16 per 100 Mb) of genes encoding for so-called Nod factor biosynthesis (**Table 2**).

Finally, each microbiome codes for a significant number (42–53 per 100 Mb) of flagellin-related genes. Since these proteins have previously been associated with triggering plant innate immunity (**Table 2**).

#### Genome Reconstruction of Alga-Associated Bacteria

To further examine the metagenome data, the assembled contigs were binned using MaxBin (Wu et al., 2014) (**Supplementary Table S2**, Bioproject number: PRJEB15376). Thereby, we established 11 high quality bins for the *C. saccharophila* microbiome, 8 for the *S. quadricauda* microbiome, and 20 bins for the *M. crux-melitensis* microbiome. These considered bins ranged from 2.8 to 11.6 Mb in size with a completeness of >70–99% (**Supplementary Table S2**). Overall the phylogenetic analyses of the bins basically reflected the population structure as determined by marker gene analysis. In the microbiome of *C. saccharophila*, the most complete bins mapped to bacteria affiliated with the following genera [genus (family)]: *Spirosoma* (Cytophagaceae), *Methylobacterium* (Methylobacteriaceae), *Mesorhizobium* (Phyllobacteriaceae), *Porphyrobacter* (Erythrobacteraceae), *Rhodococcus* (Nocardiaceae), uncultured Hyphomicrobiaceae and Rhizobiales, and uncultured Sphingomonadales. The bins with high completeness of the *S. quadricauda* microbiome were mainly affiliated with *Dyadobacter* (Cytophagaceae), *Rhodobacter sphaeroides* (Rhodobacteraceae), *Sphingopyxis* (Sphingomonadaceae), and uncultured Rhizobiales. The phylogenetic assignment of the *M. crux-melitensis* microbiome bins indicated that the bacteria were affiliated with *Phenylobacterium* (Caulobacteraceae), *Oligotropha* (Bradyrhizobiaceae), uncultured Chitinophaga, Cytophagaceae and uncultured Bacteroidetes, as well as uncultured Sphingomonadaceae, Enterobacteriaceae and uncultured Rhodospirillales. Overall, the obtained bins confirmed the overall phylogenetic structure of the communities.

### RNA-Seq Identifies Metabolically Highly Active Bacterial Groups and As Well As Highly Transcribed Genes in the Microalga Microbiomes

**FIGURE 2:**
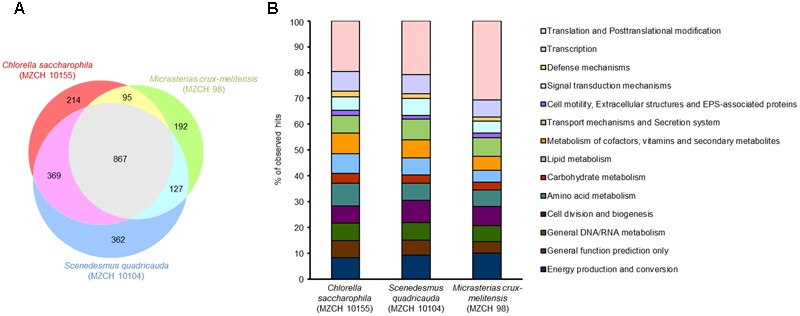
Identification of differentially expressed genes of bacterial communities of *Chlorella saccharophila* (MZCH 10155), *Scenedesmus quadricauda* (MZCH 10104), and *Micrasterias crux-melitensis* (MZCH 98) using COG-based analyses. Genes with a minimum of 10.0 Reads Per Kilobase per Million reads (RPKM) or higher where considered as expressed. **(A)** Venn diagram showing the number of genes uniquely regulated in once or commonly expressed in two or three of the microbiome. **(B)** Comparison of expression of the different bacterial communities in % of observed hits. The classification was based on the COG database.

In the light of the aforementioned findings, we asked which genes with relevance to plant–microbe interactions were highly transcribed in the alga microbiomes. In addition, we wanted to know, whether the highly transcribed genes were affiliated mainly with the α-Proteobacteria, the Bacteroidetes, or other phyla. Therefore, we established a set of RNA-Seq data for each of the three microbiomes. Nine individual samples were analyzed by RNA-Seq representing three independent biological samples for each of the three microbiomes (**Supplementary Table S3**). A minimum of 24 mio cDNA reads could be uniquely mapped for the *C. saccharophila* transcriptome dataset. More than 18 mio reads were uniquely aligned for the *S. quadricauda* cDNA and over 10 mio could be aligned for the *M. crux-melitensis* transcriptome. Genes with a RPKM value of 10.0 or higher where considered as expressed. In order to calculate the gene expression level between different communities, the level of transcribed COGs were used to group genes (**Figure 2**). For statistical analyses, the Fisher’s exact test was applied in order to detect COGs enriched in the transcriptome datasets compared to the corresponding microbiomes. Overall, microbial genes encoding ribosomal proteins, and metabolic pathways (e.g., carbohydrate-, lipid-, amino acid metabolism, and general function including energy production pathways) were among the most strongly enriched ones. In addition, we detected a high number of enriched COGs, which encoded for metabolism of cofactors, vitamins and secondary metabolites, signal transduction mechanisms, and transport mechanisms and secretion systems. The bacterial ribosomal proteins are, of course, distinct from the ribosomal rRNAs depleted from metatranscriptomic assays.

We observed that a shared set of 867 common genes were expressed in all three microbiomes (**Figure 2**). Overall, the strongest transcribed genes in all three microbiomes were genes with the following functions: transcription and translation including posttranslational modifications (24–35% of common genes), energy production and conversion, transport mechanisms and secretion system, cofactor metabolism, and unknown function (each 5–9% of common genes). However, in each alga microbiome a different set of specific genes was expressed. In the *C. saccharophila*-associated microbiome, 214 specific genes were expressed, in the *M. crux-melitensis*, 192 genes, and in the *S. quadricauda* microbiome, 362 genes (**Figure 2** and **Supplementary Table S4**).

The main difference between the three microbiomes transcripts were observed with respect to genes affiliated with transport and secretion processes, vitamin biosynthesis, signaling pathways and flagella biosynthesis. In the *C. saccharophila* transcriptome, we observed that specifically genes affiliated with the type II, IV, and VI protein secretion systems were highly transcribed. For the *S. quadricauda* transcriptome, we also identified components of the sugar-specific permease components of ABC-type transport systems (ribose, xylose, arabinose, and galactoside). Further many bacterial genes coding for proteins involved in carbohydrate and lipid modification were strongly transcribed.

**FIGURE 3:**
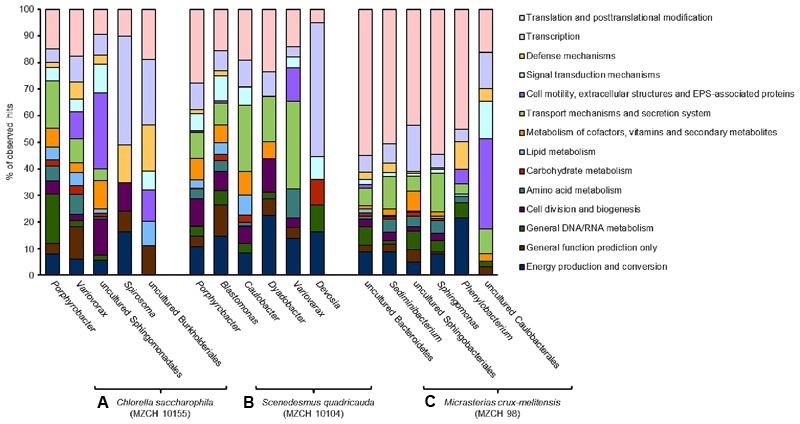
Transcribed microbiome and expressed genes of individual bacterial species. The analyses include a minimum of 50,000 reads for each bacterial species. **(A)** Bacterial community of *Chlorella saccharophila* (MZCH 10155), **(B)** bacterial community of *Scenedesmus quadricauda* (MZCH 10104), **(C)** bacterial community of *Micrasterias crux-melitensis* (MZCH 98).

Finally, in the *M. crux-melitensis* transcriptome, we identified mainly genes coding for ABC-transporters, long-chain fatty acid transporters as well as biopolymer transporter genes were highly transcribed. Interestingly, the number of genes involved in translation and posttranslational modifications were overrepresented in the transcription profile compared to the *C. saccharophila* and *S. quadricauda* microbiome (**Figure 3**).

#### *Chlorella saccharophila* Microbiome Analyses of Highly Transcribed Genes

In the *Chlorella* microbiome the majority of the 1,000 highest transcribed genes were affiliated with the α- and β-Proteobacteria. Within the α-Proteobacteria *Porphyrobacter* (Erythrobacteraceae) allowed mapping of the most transcripts (3.2 mio reads). Further, 0.8 mio reads were mapped to bacteria affiliated with the genus *Variovorax* (Comamonadaceae, β-Proteobacteria) and a large fraction of mapped reads (0.2 mio) was assigned to bacteria affiliated with the genus *Spirosoma*, belonging to the Flexibacteriacae within the Bacteroidetes phylum (**Figure 3** and **Supplementary Table S5**).

**FIGURE 4:**
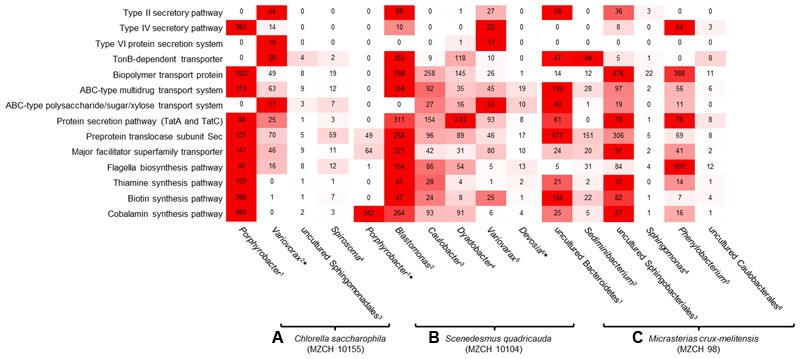
**Heatmap reflecting the expression of genes** (minimum 10.0 RPKM, color key: 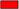 high expression level, 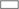 low expression level) affiliated with transport and secretion pathways, signaling molecules, cofactor, and vitamin-related enzymes of individual bacterial bins generated from the bacterial metagenomes of **(A)** *Chlorella saccharophila* (MZCH 10155) ^1^Bin-ID: 10155.001, ^2∗^Bin-ID: 10155.003, ^3^Bin-ID: 10155.011, ^4^Bin-ID: 10155.010. **(B)** *Scenedesmus quadricauda* (MZCH 10104) ^1∗^Bin-ID: 10104.004, ^2^Bin-ID: 10104.013, ^3^Bin-ID: 10104.007, ^4^Bin-ID: 10104.012, ^5^Bin-ID: 10104.018, ^6∗^Bin-ID: 10104.019 and **(C)** bacterial community of *Micrasterias crux-melitensis* (MZCH 98) ^1^Bin-ID: 98.003, ^2^Bin-ID: 98.008, ^3^Bin-ID: 98.002, ^4^Bin-ID: 98.020, ^5^Bin-ID: 98.005, ^6^Bin-ID: 98.019. The bins showed a completeness of >70–99%, low quality bins with a completeness of 25.2–66.4% are marked by ^∗^ (**Supplementary Table S2**).

Besides general cellular functions, highly transcribed genes were involved in biosynthesis and transport of vitamins. Genes involved in vitamin B12 synthesis where found in contigs and bins established for *Porphyrobacter* and *Variovorax* or other closely related organisms (**Figure 4**). This was similar for genes linked to type II, IV secretion systems and ferrochelatin transport. Most transcribed genes mapped to flagella biosynthesis are originated to *Porphyrobacter* but also Burkholderiales-related bins.

#### *Scenedesmus quadricauda* Microbiome Analyses of Highly Transcribed Genes

Similar to the *Chlorella* microbiome the most strongly transcribed genes of the *Scenedesmus* microbiome were associated with the α- and β-Proteobacteria and as well the Bacteroidetes. Of the 1,000 highest transcribed genes, 4.4 mio reads mapped on contigs with high similarity to *Porphyrobacter*. In addition, 1.1 mio reads were assigned to *Blastomonas* (Sphingomonadaceae, α-Proteobacteria). Smaller numbers of reads mapped to bins from *Caulobacter* (Caulobacteraceae, α-Proteobacteria) (0.08 mio reads), *Dyadobacter* (Cytophagaceae) within the phylum of the Bacteroidetes (0.06 mio), *Variovorax* (Comamonadaceae, β-Proteobacteria) (0.03 mio), and *Devosia* (Hyphomicrobiaceae, α-Proteobacteria) (0.03 mio) (**Figure 3** and **Supplementary Table S6**).

Most transcripts involved in vitamin B12 biosynthesis or transport mapped to *Porphyrobacter* and *Blastomonas*-related sequences. Interestingly, in this microbiome *Blastomonas* appeared to be the most active bacterium, as deduced from the number of RNA-Seq reads mapped on the secretion systems, flagella biosynthesis, and vitamin biosynthesis (**Figure 4**).

#### *Micrasterias crux-melitensis* Microbiome Analyses of Highly Transcribed Genes

In contrast to the Trebouxiophyceae and Chlorophyceae microalga in the Zygnematophyceae microbiome of *Micrasterias*, the largest fraction of the highest transcribed genes originated from bacteria affiliated with the phylum of the Bacteroidetes (1.9 mio reads). This was surprising, since the 16S rRNA and metagenome analyses had not indicated that the Bacteroidetes were represented at high levels in the microbiome (Supplementary Figure S1B and **Figure 1D**). Within the phylum of the Bacteroidetes a large fraction was assigned to sequences being highly similar to Sphingobacteria (0.5 mio reads) and on a species level 0.6 mio reads were linked to an organism highly similar to *Sediminibacterium* sp. (**Figure 3** and **Supplementary Table S7**).

In line with these observations within the *Micrasterias* affiliated microbiome, the Bacteroidetes (e.g., bins from *Sediminibacterium*, an uncultured Bacteroidetes, and *Sphingobacterium*), appeared to be the main suppliers of vitamin B12 based on the RNA-Seq data (**Figure 4**).

Altogether these data imply that in the *Chlorella* and *Scenedesmus* microbiome the bacteria affiliated with *Porphyrobacter* and *Blastomonas* are transcriptionally most active, while in the *Micrasterias* microbiome the bacteroidetal organisms show highest numbers of transcripts.

### Secretome-Based Microbiome Analysis

Secretome analyses for the *S. quadricauda* microbiome were performed to partially verify the metagenome and metatranscriptome-based results (Supplementary Figure S2 and **Table S8**).

In total, we identified 156 bacterial proteins in the supernatants. The majority of the proteins identified originated from bacteria affiliated with the α- and β-Proteobacteria. Of these, a total of 55 proteins originated most likely from *Porphyrobacter*, eight from *Blastomonas*, and six from uncultured Sphingomonadales. Furthermore, 43 proteins originated from a non-cultivated bacterium and 42 from a *Mesorhizobium* species present within the microbiome. These data concur mostly with the phylogenetic analyses, the metagenome and with the RNA-Seq analyses. However, they also indicate an important role of non-cultivated bacteria and a mesorhizobial isolate in this alga microbiome, which was not detected having significant transcriptional activities.

The functional evaluation of the protein data shows that mainly proteins were found in the supernatant that are linked to protein biosynthesis, transport systems, and hypothetical proteins. The largest fraction (41 hits) was linked to ribosomal proteins. Thirty-five hits contained proteins with no function assigned to yet. Furthermore, 28 proteins were identified as periplasmic components of ABC transporters indicating that transport of small molecules is an important key feature in this alga microbiome. Additionally, 13 outer membrane and peptidoglycan-associated proteins, seven superoxide dismutases, seven proteins involved amino acid biosynthesis/modifications, five flagellins, and few hits for cobalamin and TonB receptor proteins were identified.

## Discussion

Microalga affiliated with the phylum of the chlorophytes and the charophytes are the ancestors of higher land plants. The term microalga comprises a phylogenetically very heterogeneous group of pro- and eukaryotic microorganisms with global occurrence. They all employ oxygenic photosynthesis and play an important role in the global carbon cycling and oxygen production (Falkowski and Raven, 2013). While for land plants, intensive studies have been undertaken to investigate the phylogeny and role of either associated, endophytic, or symbiotic bacteria, only very few studies exist, which have addressed a phylogenetic analyses of microalga microbiota including tightly attached phycosphere biofilms (Lakaniemi et al., 2012; Krohn-Molt et al., 2013; Mendes et al., 2013; Krustok et al., 2015; Cúcio et al., 2016; Ramanan et al., 2016).

With respect to the phylogenetic analyses, bacteria observed in alga affiliated microbiomes belong to the α-, β-, and γ-Proteobacteria. In addition, bacteria affiliated with the phylum of the Bacteroidetes are frequently observed. Furthermore, many uncultivated bacteria are noted, and no archaeal species have yet been detected (Krohn-Molt et al., 2013; Knack et al., 2015; Krustok et al., 2015; Ramanan et al., 2016). Thus, these data are in line with our observations. However, and in addition to the previous studies, our data imply that high levels of specificity exist with respect to the phylogeny. This was the case for the microbiomes of the three species of the microalga affiliated with the genus *Scenedesmus* and *Micrasterias*. Lower levels of specificity appeared to exist for *Chlorella*-associated microbiomes (Supplementary Figure S1 and **Figure 1D**). Furthermore, we not only rely on 16S rRNA gene analyses but performed almost complete metagenome sequencing analyses including phylogenetic analysis of additional 31 marker genes as well as an assessment of the functional repertoire of the microbiome. In addition, many of the generated bins of the metagenome datasets represent known root, plant or host-associated bacteria. With respect to the plant–bacteria signaling pathways, we observed rather a low number of AI biosynthesis proteins in the metagenome datasets. This may indicate a minor role of these classical AI signaling pathways in the microbiome communities. In contrast, we hypothesize that flagellin-related genes are already of relevance in this evolutionary early plant–microbe interaction (**Table 2**).

Our transcriptome data indicated that in general very common processes are expressed in all three microbiomes with differences mainly in few host interaction-related processes (**Figure 2**). Surprisingly, many of the transcripts matched genes that are known to be of relevance in the interaction of higher plants. These included the infection-related secretion pathways, biosynthesis of exoenzymes, modifying of carbohydrates and lipids and flagella biosynthesis pathways (**Table 2** and **Figure 4**). Especially the occurrence of flagella-related proteins was confirmed by additional secretome studies. Mainly bacterial secretion systems type II, III, IV, and VI have been shown to be of relevance for plant–microbe interaction processes (Gophna et al., 2003; Alvarez-Martinez and Christie, 2009; Nivaskumar and Francetic, 2014; Zoued et al., 2014). Similarly, flagella are well known for their role in plant infection and inducing plant innate immunity (Jones and Dangl, 2006). Within this framework, our findings imply that at least some of the triggers and signals involved in the microbial interaction with higher plants are already of relevance in this evolutionary early system (**Figures 3, 4**).

Furthermore, microalgae are in general auxotrophic for vitamin B12. Additionally, a significant fraction of algae species is auxotrophic for thiamine and some are also auxotrophic for biotin (Warren et al., 2002; Helliwell et al., 2011; Giovannoni, 2012). Therefore, one of the main benefits of the bacterial interaction is the supply with the needed B-vitamins. While it has previously been reported that alga-associated bacteria are most likely responsible for the supply of the essential B-vitamins, we provide strong evidence that the supply is provided by a rather small group of bacteria in the different microbiomes and that significant differences exist between the *Chlorella, Scenedesmus*, and *Micrasterias* associated bacterial community (**Figure 4**). While mostly the α-Proteobacteria (e.g., *Porphyrobacter* and the *Blastomonas*) appeared to be the main suppliers of B-vitamins in the *Chlorella*, and *Scenedesmus* microbiomes, in the *Micrasterias* microbiome, uncultivated bacteroidetal species and Sphingobacteria shows higher numbers of reads on a transcriptional level (**Figure 4**) with respect to the biosynthesis of B12.

In summary, the current study gives a detailed insight into the almost complete metagenome, transcriptome, and partial secretome of three microalga. Our findings support the idea that phylogenetic different bacterial groups are of relevance in the selected microalga microbiomes. Finally, our study shows that microalga–bacteria microbiomes are relevant models to study early evolutionary plant–microbe interactions. While this study has been done with alga-microbiomes that originated from strain collections future work will now address the analysis of more complex systems in native phytoplankton samples.

## Author Contributions

IK-M contributed to experimental design; lab work of phylogenetic, metagenomic, transcriptomic, and proteomic approaches; and writing of the research article. MA contributed to assembly of metagenomic datasets and bioinformatic approaches. KF contributed to assembly of transcriptomic datasets and bioinformatic approaches. AW contributed to lab work of proteomic approaches. LB and DI contributed to lab work of metagenomic and phylogenetic approaches. MT contributed to lab work of proteomic approaches. AG contributed to experimental design of phylogenetic, metagenomic, and bioinformatic approaches. JK and AT contributed to experimental design of proteomic approaches. WS contributed to general experimental design and writing of the research article.

## Conflict of Interest Statement

The authors declare that the research was conducted in the absence of any commercial or financial relationships that could be construed as a potential conflict of interest.

## Footnotes

1 https://www.biologie.uni-hamburg.de/biozentrum-klein-flottbek/forschung/aoepp-hanelt/algensammlung.html

2 https://www.illumina.com

3 http://www.vertis-biotech.com/

4 http://hannonlab.cshl.edu/fastx_toolkit/

5 https://doi.org/10.5281/zenodo.848556

6 http://www.iem.uni-kiel.de/de/systematische-proteomics

7 http://www.ebi.ac.uk/ena/data/view/PRJEB15376

8 https://img.jgi.doe.gov

## Supplementary Material

TABLE S1 Overall number of contigs generated for bacterial community of *C. saccharophila* (MZCH 10155), *Scenedesmus quadricauda* (MZCH 10104) and *Micrasterias crux-melitensis* (MZCH 98), including protein coding genes and data source.

TABLE S2 Bin overview of the bacterial community of *C. saccharophila* (MZCH 10155), *S. quadricauda* (MZCH 10104), and *M. crux-melitensis* (MZCH 98) including phylogenetic assignment (consensus score > 0.8), completeness (mainly mine cut off 70.0%, ^*^low quality bins 25.2-66.4%), genome size and GC content.

TABLE S3 RNA-seq processing, number of cDNA sequence reads obtained for bacterial community of *C. saccharophila* (MZCH 10155), *S. quadricauda* (MZCH 10104), and *M. crux-melitensis* (MZCH 98). The results represent the average value of 3 replicas.

TABLE S4 Overview transcriptomic assignment including function, COGs and counting of the RNA seq data of *C. saccharophila* (MZCH 10155), *S. quadricauda* (MZCH 10104), and *M. crux-melitensis* (MZCH 98), minimum RPKM value: 10.0.

TABLE S5 Transcriptomic assignment of the first most abundant 1000 hits of the *C. saccharophila* (MZCH 10155) RNA seq dataset including functions and pathways.

TABLE S6 Transcriptomic assignment of the first most abundant 1000 hits of the *S. quadricauda* (MZCH 10104) RNA seq dataset including functions and pathways.

TABLE S7 Transcriptomic assignment of the first most abundant 1000 hits of the *M. crux-melitensis* (MZCH 98) including functions and pathways.

TABLE S8 List of proteins observed in the common elements of LC/MS-MS analysis of *S. quadricauda* (MZCH 10104) including the overlap between secretome and transcriptome datasets and phylogenetic assignment.
