# Supplementary material for: Insights into Microalga and Bacteria Interactions of Selected Phycosphere Biofilms Using Metagenomic, Transcriptomic, and Proteomic Approaches
Source: Front Microbiol. 2017 Oct 10;8:1941. doi: 10.3389/fmicb.2017.01941 (PMC5641341; doi:10.3389/fmicb.2017.01941)
Supplement: TABLE S1 — Overall number of contigs generated for bacterial community of C. saccharophila (MZCH 10155), Scenedesmus quadricauda (MZCH 10104) and Micrasterias crux-melitensis (MZCH 98), including protein coding genes and data source. [file Table_1.docx]

# Supplemental TABLES

TABLE S1: Overall number of contigs generated for bacterial community of Chlorella saccharophila (MZCH 10155), Scenedesmus quadricauda (MZCH 10104) and Micrasterias crux-melitensis (MZCH 98), including protein coding genes and data source.

|  | *Chlorella saccharophila*  (MZCH 10155) | *Scenedesmus quadricauda*  (MZCH 10104) | *Micrasterias crux-melitensis*  (MZCH 98) |
| --- | --- | --- | --- |
| **Number** | 82,149 | 50,727 | 120,444 |
| **Total length (bp)** | 174,776,540 | 162,329,183 | 268,164,566 |
| **Number > = 1000 bp** | 39,300 | 21,396 | 48,039 |
| **N50 size (bp)** | 3,620 | 13,604 | 6,026 |
| **Largest (bp)** | 848,424 | 270,578 | 1,553,612 |
| **GC mean (%)** | 63.28 | 58.41 | 61.88 |
| **Protein coding genes** | 236,273 | 270,387 | 349,091 |
| **Data source** | IMG ID 3300008885 | IMG ID 3300005759 | IMG ID 3300008886 |
