# Supplementary material for: Insights into Microalga and Bacteria Interactions of Selected Phycosphere Biofilms Using Metagenomic, Transcriptomic, and Proteomic Approaches
Source: Front Microbiol. 2017 Oct 10;8:1941. doi: 10.3389/fmicb.2017.01941 (PMC5641341; doi:10.3389/fmicb.2017.01941)
Supplement: TABLE S2 — Bin overview of the bacterial community of C. saccharophila (MZCH 10155), S. quadricauda (MZCH 10104), and M. crux-melitensis (MZCH 98) including phylogenetic assignment (consensus score > 0.8), completeness (mainly mine cut off 70.0%, *low quality bins 25.2-66.4%), genome size and GC content. [file Table_2.docx]

# Supplemental TABLES

TABLE S2: Bin overview of the bacterial community of Chlorella saccharophila (MZCH 10155), Scenedesmus quadricauda (MZCH 10104) and Micrasterias crux-melitensis (MZCH 98) including phylogenetic assignment (consensus score > 0.8), completeness (mainly min cut off 70.0%, *low quality bins 25.2-66.4%), genome size and GC content.

|  | Bin-ID | Complete-ness  (%) | Genome size  (bp) | GC content  (mol%) |
| --- | --- | --- | --- | --- |
| **bacterial community of *Chlorella saccharophila* (MZCH 10155)** | | | | |
| Bin affiliated to: |  |  |  |  |
| ***Porphyrobacter*** (Erythrobacteraceae) | 10155.001 | 90.7 | 2,846,376 | 65.7 |
| ***Methylobacterium*** (Methylobacteriaceae) | 10155.007 | 99.1 | 5,646,795 | 66.3 |
| ***Sinorhizobium*** (Rhizobiaceae) | 10155.008 | 97.2 | 5,684,290 | 60.3 |
| uncultured **Hyphomicrobiaceae** | 10155.009 | 91.6 | 5,435,896 | 65.4 |
| ***Spirosoma linguale*** (Cytophagaceae) | 10155.010 | 99.1 | 6,955,678 | 55.7 |
| uncultured **Sphingomonadales** | 10155.011 | 96.3 | 3,984,042 | 63.2 |
| ***Mesorhizobium ciceri*** (Phyllobacteriaceae) | 10155.012 | 98.1 | 8,099,946 | 62.4 |
| uncultured **Rhizobiales** | 10155.013 | 99.1 | 9,705,004 | 61.1 |
| ***Rhodococcus erythropolis*** (Nocardiaceae) | 10155.019 | 94.4 | 7,052,564 | 62.4 |
| ***Cytophaga hutchinsonii*** (Cytophagaceae) | 10155.020 | 75.7 | 3,437,315 | 59.8 |
| uncultured **Proteobacteria** | 10155.021 | 88.8 | 5,946,441 | 54.1 |
| ***Variovorax*** (Comamonadaceae)***** | 10155.003 | 66.4 | 2,182,774 | 67.2 |
| **bacterial community of *Scenedesmus quadricauda* (MZCH 10104)** | | | | |
| Bin affiliated to: |  |  |  |  |
| ***Sphingopyxis*** (Sphingomonadaceae) | 10104.002 | 95.3 | 6,214,307 | 65.7 |
| ***Caulobacter* sp. K31** (Caulobacteraceae) | 10104.007 | 94.4 | 4,346,074 | 65.9 |
| ***Rhodobacter sphaeroides*** (Rhodobacteraceae) | 10104.008 | 96.3 | 4,367,578 | 65.2 |
| ***Mesorhizobium*** (Phyllobacteriaceae) | 10104.009 | 82.2 | 5,260,893 | 63.8 |
| ***Dyadobacter fermentans*** (Cytophagaceae) | 10104.012 | 99.1 | 8,849,123 | 45.2 |
| uncultured **Sphingomonadaceae** | 10104.013 | 81.3 | 3,194,051 | 64.7 |
| uncultured **Rhizobiales** | 10104.017 | 98.1 | 5,182,423 | 65.3 |
| ***Variovorax*** (Comamonadaceae) | 10104.018 | 78.5 | 6,005,369 | 67.5 |
| ***Porphyrobacter*** (Erythrobacteraceae)* | 10104.004 | 25.2 | 1,186,823 | 64.0 |
| ***Devosia*** (Hyphomicrobiaceae)* | 10104.019 | 63.6 | 4,863,421 | 67.0 |
| **bacterial community of *Micrasterias crux-melitensis* (MZCH 98)** | | | | |
| Bin affiliated to: |  |  |  |  |
| uncultured **Chitinophaga** | 98.001 | 95.3 | 3,566,736 | 45.5 |
| uncultured **Sphingomonadaceae** | 98.002 | 97.2 | 4,629,653 | 64.4 |
| uncultured **Bacteroidetes** | 98.003 | 98.1 | 6,287,032 | 46.7 |
| uncultured Rhodospirillales | 98.004 | 99.1 | 6,598,965 | 67.4 |
| ***Phenylobacterium*** (Caulobacteraceae) | 98.005 | 97.2 | 5,330,430 | 68.8 |
| *Sediminibacterium* (Chitinophaga) | 98.008 | 93.5 | 7,471,094 | 50.5 |
| uncultured Proteobacteria | 98.009 | 96.3 | 7,697,153 | 65.6 |
| *Oligotropha* (Bradyrhizobiaceae) | 98.012 | 97.2 | 3,729,856 | 60.3 |
| uncultured Rhizobiales | 98.013 | 76.6 | 4,523,610 | 67.7 |
| *Isosphaera pallida* (Planctomycetaceae) | 98.015 | 89.7 | 9,139,317 | 66.0 |
| uncultured Caulobacteraceae | 98.019 | 91.6 | 4,389,302 | 67.7 |
| *Sphingomonas* (Sphingomonadaceae) | 98.020 | 72.0 | 4,175,088 | 63.8 |
| *Mesorhizobium* (Phyllobacteriaceae) | 98.022 | 92.5 | 11,619,093 | 61.8 |
| uncultured Bacteria | 98.023 | 92.5 | 3,776,644 | 35.7 |
| uncultured Enterobacteriaceae | 98.026 | 99.1 | 4,446,778 | 51.1 |
| uncultured Cytophagaceae | 98.027 | 95.3 | 5,094,132 | 61.1 |
| uncultured Alphaproteobacteria | 98.028 | 70.1 | 7,626,695 | 65.6 |
| *Acidimicrobium* (Acidimicrobiaceae) | 98.031 | 75.7 | 3,819,907 | 69.1 |
| *Ralstonia pickettii* (Burkholderiaceae) | 98.034 | 76.6 | 7,124,889 | 64.0 |
| uncultured Rhizobiales | 98.040 | 72.9 | 6,002,861 | 62.1 |
