# Supplementary material for: Insights into Microalga and Bacteria Interactions of Selected Phycosphere Biofilms Using Metagenomic, Transcriptomic, and Proteomic Approaches
Source: Front Microbiol. 2017 Oct 10;8:1941. doi: 10.3389/fmicb.2017.01941 (PMC5641341; doi:10.3389/fmicb.2017.01941)
Supplement: TABLE S3 — RNA-seq processing, number of cDNA sequence reads obtained for bacterial community of C. saccharophila (MZCH 10155), S. quadricauda (MZCH 10104), and M. crux-melitensis (MZCH 98). The results represent the average value of 3 replicas. [file Table_3.docx]

# Supplemental TABLES

TABLE S3: RNA-seq processing, number of cDNA sequence reads obtained for bacterial community of Chlorella saccharophila (MZCH 10155), Scenedesmus quadricauda (MZCH 10104) and Micrasterias crux-melitensis (MZCH 98). The results represent the average value of 3 replicas.

|  | Chlorella saccharophila  (MZCH 10155) | Scenedesmus quadricauda  (MZCH 10104) | *Micrasterias crux-melitensis*  (MZCH 98) |
| --- | --- | --- | --- |
| **Number of reads generated** | 28,422,068 | 21,403,378 | 15,890,586 |
| **Total number of aligned reads** | 26,761,874 | 19,172,194 | 13,278,587 |
| **Total number of uniquely aligned reads** | 24,760,057 | 18,863,417 | 10,970,434 |
| **Processed reads length distribution (nt, Mean calculation)** | 71.34 | 72.34 | 71.12 |
| **Number of genes with sufficiently high RPKM (10.0)** | 5,198 | 7,603 | 7,764 |
