# Supplementary material for: Insights into Microalga and Bacteria Interactions of Selected Phycosphere Biofilms Using Metagenomic, Transcriptomic, and Proteomic Approaches
Source: Front Microbiol. 2017 Oct 10;8:1941. doi: 10.3389/fmicb.2017.01941 (PMC5641341; doi:10.3389/fmicb.2017.01941)
Supplement: Supplementary file 9 [file Image_1.pdf]

Supplemental FIGURES

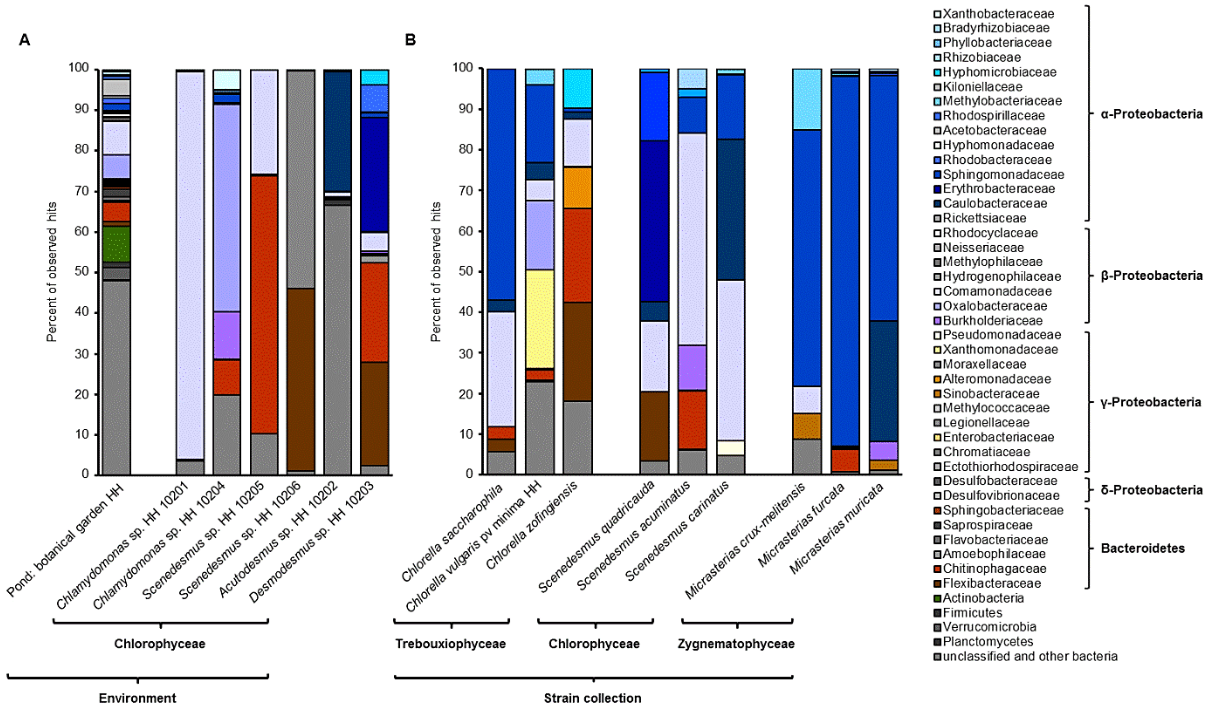

FIGURE S1: Identification and bacterial distribution of dominant bacterial families via 16S rRNA amplicon based phlyogenetic analysis attached to various microalgae from (A) fresh water environment (pond "Botanical Garden" of the University of Hamburg, Germany), and (B) samples of the microalgae strain collection of Hamburg.
