# Supplementary material for: Insights into Microalga and Bacteria Interactions of Selected Phycosphere Biofilms Using Metagenomic, Transcriptomic, and Proteomic Approaches
Source: Front Microbiol. 2017 Oct 10;8:1941. doi: 10.3389/fmicb.2017.01941 (PMC5641341; doi:10.3389/fmicb.2017.01941)
Supplement: Supplementary file 10 [file Image_2.pdf]

Supplemental FIGURES

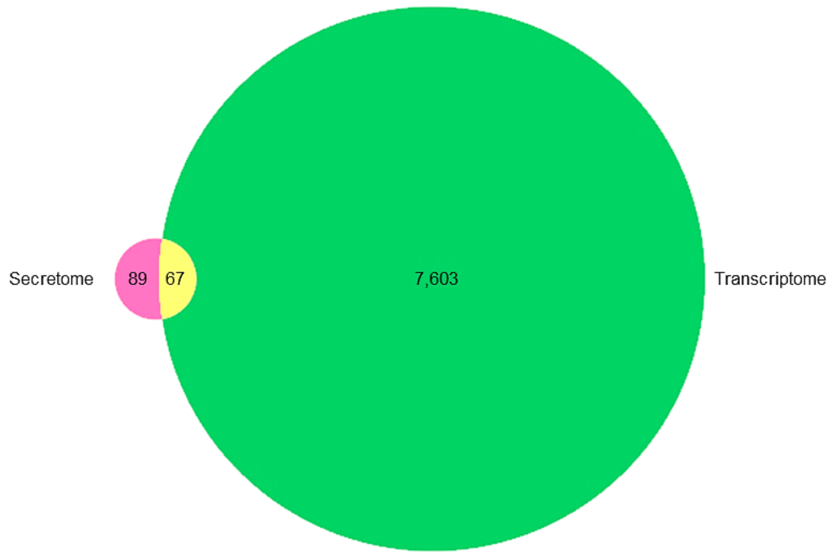

FIGURE S2: Venn diagram showing the number of uniquely regulated genes of the transcriptome-dataset (RPKM 10.0, green) versus the expressed proteins identified by the secretome-dataset (red) of *Scenedesmus quadricauda* (MZCH 10104).
